# Effect of Various Vital Bleaching Systems on Clinical Outcomes and Patient Satisfaction

**DOI:** 10.7759/cureus.65648

**Published:** 2024-07-29

**Authors:** Mohamed Samir A Elnawawy, Harshkant Gharote, Fawaz Pullishery, Rehab Al Wakeb, Basem Abuzenada

**Affiliations:** 1 Clinical Sciences Department, General Dentistry Program, Batterjee Medical College, Jeddah, SAU; 2 Conservative Dentistry, Mansoura University, Mansoura, EGY; 3 Dental Public Health and Research Department, Batterjee Medical College, Jeddah, SAU; 4 Clinical Sciences Department, Batterjee Medical College, Jeddah, SAU; 5 Restorative Dentistry Department, King Abdulaziz University, Jeddah, SAU

**Keywords:** color change, gingival irritation, dental sensitivity, carbamide peroxide, titanium dioxide, hydrogen peroxide

## Abstract

Objective

To evaluate the clinical behavior of different bleaching products - the hydrogen peroxide (H_2_O_2_), carbamide peroxide (CP), and titanium dioxide bleaching systems.

Methods

Three bleaching systems with different concentrations (H_2_O_2_ 15%, 38%, CP 15%, 35%, and titanium dioxide 20% H_2_O_2_) were used. Sixty participants with discolored teeth were enrolled and equally divided into six groups. Each group was rendered the assigned bleaching protocol against the control group, which received plain dentifrice polishing. Each case was evaluated immediately, two weeks, three months, six months, and one year after the bleaching treatment. The clinical evaluation was made for color change by (shade guide and digital images with L*a*b* parameters), tooth sensitivity, gingival irritation, and participants' satisfaction.

Results

All bleaching systems showed color improvement after bleaching regimens with significant effect showed by in-office titanium dioxide 20% H_2_O_2_ followed by 38% H_2_O_2_ with Δ=10.26 and Δ=6.52, respectively, when compared to other bleaching techniques. Higher sensitivity was recorded in group III with 50% of the participants reporting postoperative sensitivity. Higher gingival irritation was recorded in group IV (15% CP) where 60% of the patients reported gingival irritation. Thirty-five of the 60 participants (58.5%) recorded that the treatment whitened their teeth “moderately” and “a lot” while seven participants recorded “a slight” difference. There was a highly significant difference in participants’ satisfaction between all bleaching groups (p < 0.05).

Conclusion

It is evident that there is a development of dental sensitivity and gingival irritation irrespective of the bleaching system used. The color assessment showed that the desired result can be achieved with variable levels of patient satisfaction with excellent overall results with the titanium dioxide system.

## Introduction

Tooth color is an important issue not only for the professional, for the selection of the desired tooth shade for aesthetic restorations or tooth bleaching procedures but also for patients and consumers who wish to enhance their smiles [1]. Tooth color is influenced by a combination of intrinsic color and the presence of extrinsic stains that may form on the tooth surface [2,3]. Tooth discoloration is a result of complex physical and chemical interactions between stain-causing materials and teeth and is classified as extrinsic and intrinsic stains [4].

In recent years, teeth bleaching has emerged as one of the most rapidly growing oral care sectors, fueled by the patients’ demand for both a healthy and an esthetically appealing appearance. This is because, for most people, tooth appearance is one of the most important aspects of facial attractiveness, but it could be compromised by any discoloration or staining. Presently, three kinds of tooth-bleaching techniques are available: walking bleach, in-office bleach, and at-home bleach. Amongst these, in-office bleach and at-home bleach can be used to improve the color of discolored vital teeth. Thus, they inevitably became the popular choice for removing intrinsic enamel stains, to the end of improving the esthetic appearance of teeth [5].

Despite the demonstrated safety of dental bleaching, patients are still concerned about the potential harmful effects of these treatments such as increased tooth sensitivity and gingival irritation. Transient tooth sensitivity during and after treatment was stated in approximately two-thirds of patients undergoing bleaching. This drawback has compelled investigators to search for an effective method of in-office bleaching agent with improved protection for both the teeth and soft oral tissues [6-10]. With this viewpoint, this study aimed to evaluate the clinical behavior of different bleaching systems for color change, tooth sensitivity, gingival irritation, and patient satisfaction. The color changes were evaluated primarily by use of a shade guide and clinical images were recorded using a digital camera and stored in Adobe Photoshop software (Adobe Inc., San Jose, California, USA). However, it was used only for reference purposes, and the comparison between two modalities, namely, Shade Guide and Photoshop, was not conducted. The structured questionnaires were adapted for the assessment of tooth sensitivity, gingival irritation, and patient satisfaction.

## Materials and methods

The present study was conducted on students and employees of the Faculty of Dentistry, Mansoura University, Egypt, at the Department of Conservative Dentistry, after obtaining ethical approval from the Institutional Ethical Committee.

Clinical evaluation test

Sixty participants with six maxillary anterior teeth with A3 or darker shades on the Vita Classic Shade Guide (VITA Zahnfabrik H. Rauter, GMBH & Co. KG, Germany) between the age range of 18 and 35 years were selected. Those who agreed to regular follow-up, to have sound six maxillary anterior teeth with A3 or darker on the Vita Classic Shade Guide, and to refrain from the use of tobacco products were included in the study. The exclusion criteria covered underlying medical conditions, pregnancy, lactation, and use of tobacco products during the last 30 days before the study. Further, participants with oral mucosal lesions, a gingival index score greater than 1.0, intrinsic staining, and undergoing any professional “in-office” or “at-home” bleaching were excluded from the study.

Each participant was informed of and agreed with the objectives, benefits, and possible hazards involved in the study. Sixty participants in this study were randomized and divided into six groups (10 participants each) and all the bleaching procedures were performed one week after the oral prophylaxis.

Bleaching procedures

All participants received prophylaxis paste one week before bleaching.

Group I (Control Group), Non-bleach Treated

The teeth of 10 participants received dentifrice slurry (Topex, Sultan Healthcare, New Jersey, USA) treatment only.

Group II

**Figure 1:**
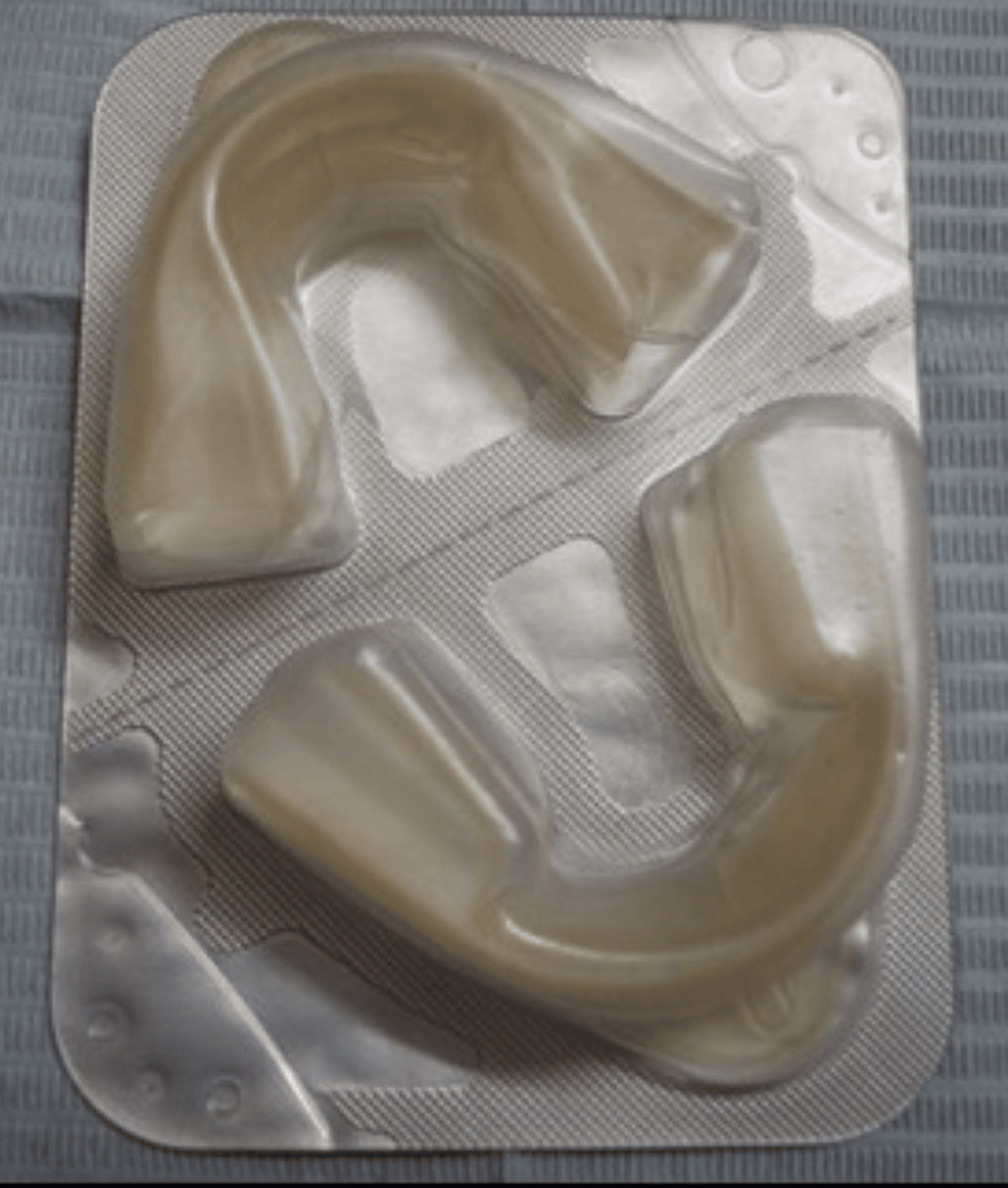
Opalescence Trèswhite Supreme 15% hydrogen peroxide (pre-loaded tray)

Six anterior teeth of 10 participants were bleached with pre-loaded whitening trays with 15% H_2_O_2_ (Opalescence Trèswhite Supreme, Ultradent Products, Inc., Utah, USA). The participants were instructed to wear the tray for 15-20 minutes per day for 10 days according to manufacturers’ instructions (Figure 1).

Group III

**Figure 2:**
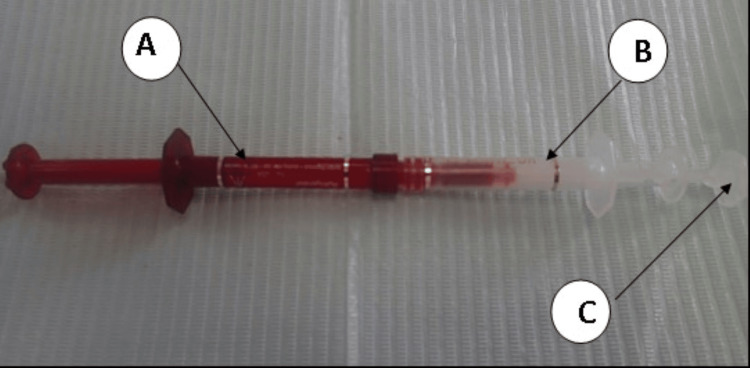
Opalescence boost 38% hydrogen peroxide A. Red Syringe, B. Clear Syringe, C. Clear Plunger

**Figure 3:**
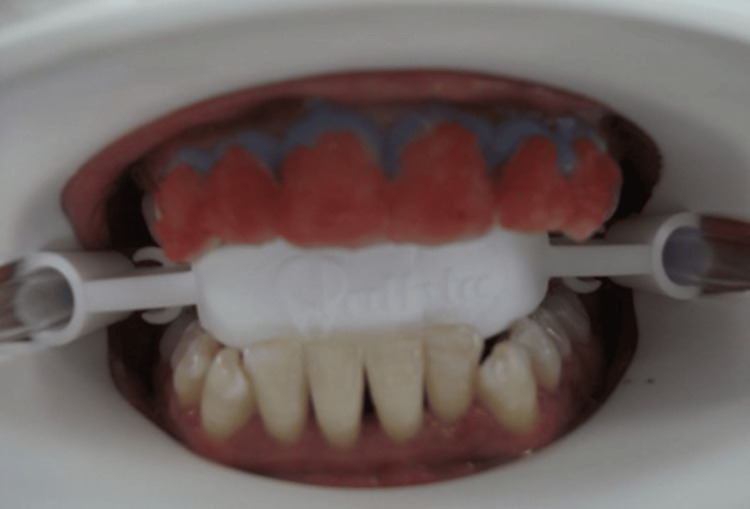
Application of opalescence boost

Six anterior teeth of 10 participants received an in-office bleaching system containing 38% H_2_O_2 _(Opalescence Boost 38%, Ultradent Products) after gingival isolation with OpalDam Green to protect gingiva, including the papilla. A thick layer of gel was applied to the labial surface of each tooth and slightly onto the incisal/occlusal surfaces and was allowed to remain on the teeth for 20 minutes as per the manufacturer’s instructions. If additional whitening was desired and no significant sensitivity was noted, participants were rescheduled every 3-5 days to repeat treatment. The procedure was performed up to two times per visit (Figures 2, 3).

Group IV

**Figure 4:**
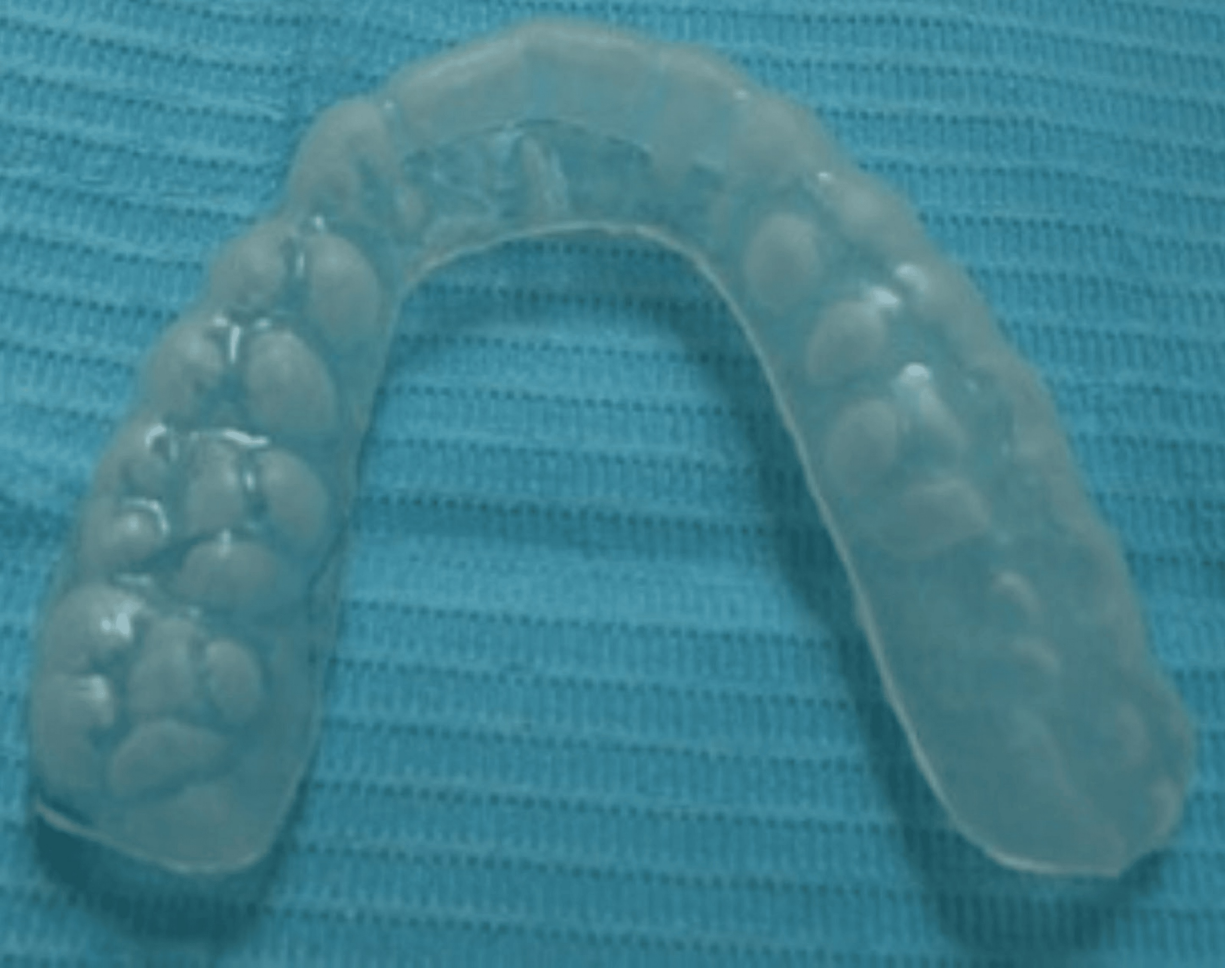
Night-guard bleaching tray after trimming

Six anterior teeth of 10 participants received the home bleach protocol with 15% carbamide peroxide (Opalescence PF 15%, Ultradent Products). All participants were provided with custom reservoir trays with scalloped labial and lingual surfaces just incisal to the free gingival margin to prevent gingival irritation. The participants were directed to fix the tray every day for four to six hours for 10 days according to manufacturers’ instructions (Figure 4).

Group V

Six anterior teeth of 10 participants were subjected to home bleaching with 35% carbamide peroxide with the same procedures in group IV with a bleaching time of 30-40 minutes per day.

Group VI

**Figure 5:**
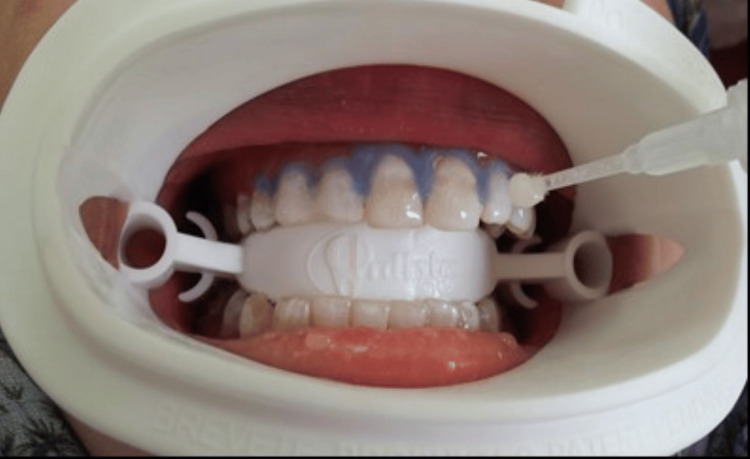
Application of GC Ti-ON whitening gel Ti-ON: in-office titanium dioxide

**Figure 6:**
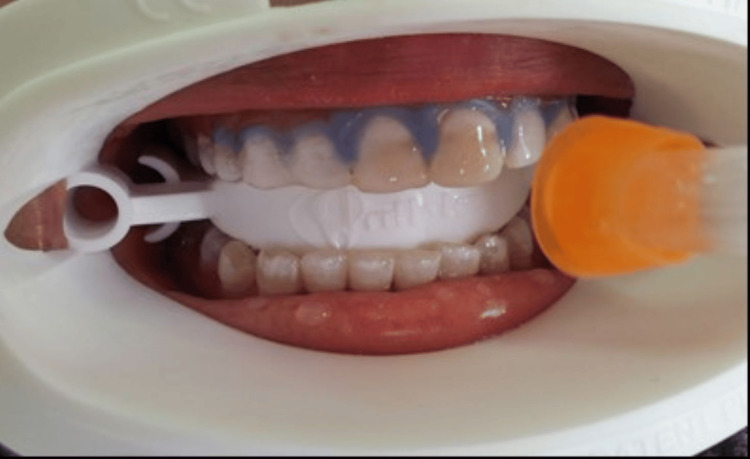
Irradiation of whitening gel

Six anterior teeth of 10 participants received in-office bleaching with 20% H_2_O_2_ (GC America Inc., Alsip, IL, USA). Soft tissue protection was achieved with petrolatum jelly for lips and titanium dioxide protector for the labial gingiva of six maxillary anterior teeth. A thin layer of reactor was applied to the surface to be whitened with a disposable tip brush and the excess was removed with an air syringe. The thoroughly mixed whitening gel was applied and light-cured by placing the source close to the tooth surface for one minute for each tooth (Figures 5, 6).

Participant’s evaluation

Each case was evaluated immediately (baseline), two weeks, three months, six months, and one year after bleaching treatment (follow-up periods) for color assessment, tooth sensitivity, gingival inflammation, and patient satisfaction.

The calibration for color assessment was done on six sound anterior teeth of two patients without bleaching as per the manufacturer's instructions. Two Operative Dentistry specialists working at Mansoura University were recruited for the color assessment with the following method:

Visual evaluation (color scale) by shade guide: The tooth color shade of all anterior teeth was recorded using the VITA Pan 3D-Master Shade Guide before and after the bleaching at determined follow-up intervals for each group. Baseline values for the initial tooth color were recorded one week after prophylaxis during the calibration phase with the use of the shade guide, which assigns a value to the color in units in descending order from lightest to darkest as set out by the manufacturer [11]. The shade guide was held with the arm bent (distance of approx. 25-30 cm), directly in front of the patient's tooth. Tooth shade was determined under natural daylight and the surrounding area was as color-neutral as possible. Female participants were asked to remove lipstick or cosmetics. The shade sample tooth was held parallel to the patient’s tooth and as close as possible to the gums and the shade of the sample tooth was found especially through the central area of the shade sample. The operator made a shade choice as early as possible and accepted the first decision since the eyes began to tire after approximately 5-7 seconds. The advantages of using the shade guide method include simplicity, ease of shade discerning, and reaching a consensus by evaluators; however, researchers have pointed out its inherent flaws as the lack of uniformity and limited color range coverage of natural teeth [11].

Color measurement by digital image capturing and analysis: A high-resolution digital camera (Fuji Film Corporation Digital Camera Finepix AV100, Shanghai, China) connected to a computer was used to have standardized digital images of anterior incisors. The images of the teeth were captured under standard illumination and constant distance. All individuals were in the same position in a single operatory with the camera positioned 9 inches and perpendicular to the tooth surface of the left central incisor. Patients were asked to touch the tongue to the soft palate after placement of the retractor. This allowed the light to readily pass through the tooth surface and reduce reflectance from their tongue. The images were evaluated using Photoshop 7.02 (Adobe Inc.). A constant point without light reflection was chosen for color evaluation. The images were further subject to the CIELAB system for assessment of color change as follows:

The CIELAB system (CIE L*a*b*) was used to assess the color difference before and after bleaching where the L* value indicates the degree of lightness, the a* value represents the degree of greenness, and the b* value detects the degree of blueness of the color. For color results, the difference between L*, a*, and b* were expressed as ΔL*, Δa*, and Δb*, where ΔL* = L*final − L*baseline, Δa* = a*final − a*baseline and Δb* = b*final − b*baseline. The overall color difference ΔE of each specimen was calculated by the expression: ΔE = [(ΔL*)2 + (Δa*)2 + (Δb*)2]1/2 [12]. The main advantage of CIELAB is that it is designed to approximate human vision where the L* component closely matches human perception of lightness.

However, the use of two methods for color assessment was planned independently for statistical analysis of the color change to confirm the results and not to compare between the two evaluation methods.

Dental sensitivity, gingival irritation, and participants' satisfaction

Tooth sensitivity was verified with a light air jet over the labial surface of the teeth. The degree of sensitivity was recorded using the criteria no sensitivity, slight sensitivity, moderate sensitivity, and severe sensitivity [13]. Potassium nitrate products, such as Ultra EZ (Ultradent), were used to manage moderate and severe sensitivity. The gingival irritation was assessed by the Löe and Silness gingival index for inflammation and was given a score from 0 to 3 (0 - normal gingiva, 1 - mild inflammation: a slight change in color and slight edema without bleeding on probing, 2 - moderate inflammation: redness, edema, and glazing and bleeding on probing, and 3 - severe inflammation with marked redness and edema, ulceration, and spontaneous bleeding).

Participants’ satisfaction was obtained after the completion of the bleaching treatment through a questionnaire using the scale: none, slight, moderate, or high. Participants were also asked if they would recommend the bleaching treatment to others using the criteria: “yes”, “maybe”, and “no” [13].

Statistical analysis

Statistical analysis of the data was done by using Statistical Package for Social Science (SPSS) version 17.0 (SPSS Inc., Chicago, IL, USA). Quantitative data were expressed as mean± SD while qualitative data were expressed as no and percentage. For parametric data, comparisons for two related groups were carried out by the paired t-test and comparisons for more than two groups were carried out by analysis of variance (ANOVA). Pearson's correlation was used to assess the relation between variables. Significance was considered when the P-value is < 0.05. All graphic representations of the data were performed with Microsoft Excel for Windows (Microsoft Corporation, Redmond, WA, USA).

## Results

Each case was followed up at the baseline and the two-week, three-month, six-month, and one-year intervals after bleaching treatment for color assessment, tooth sensitivity evaluation, gingival irritation, and participants' satisfaction. The means and standard deviations (SD) for color assessments using the shade guide and digital image capturing were recorded and tabulated for each group at various intervals, along with the overall color difference.

**Figure 7:**
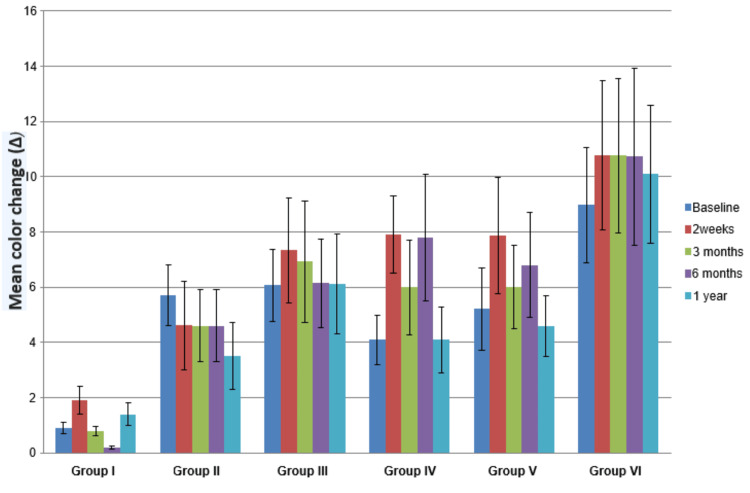
Color change Δ (shade guide)

The results of the shade guide-based assessment showed a highly significant difference in all intervals between all the groups. This revealed the in-office titanium dioxide (Ti-ON) 20% H_2_O_2_ bleaching technique has a significant bleaching effect at all intervals when compared to other bleaching techniques. The results showed that there was a significant improvement in the color of the teeth bleached with this technique. Further, the in-office Ti-ON 20% H_2_O_2_ bleaching technique followed by 38% H_2_O_2_ has a significant bleaching effect (Δ10.26 and Δ6.52) when compared to other bleaching techniques (Table 1, Figure 7).

**Table 1:** Color change (Δ) for all the bleaching groups at various intervals and the overall effect of visual evaluation for color change by a shade guide *SD: standard deviation

	Color change (Δ) for all the bleaching groups (Mean ± SD)						ANOVA	
Interval	Group I	Group II	Group III	Group IV	Group V	Group VI	F	p-value
Baseline	0.9±0.2	5.71±1.1	6.07±1.3	4.09±0.9	5.21±1.5	8.97±2.1	40.28	0.000
2 weeks	1.9±0.5	4.62±1.6	7.34±1.9	7.91±1.4	7.87±2.1	10.77±2.7	28.19	0.000
3 months	0.8±0.17	4.6±1.3	6.94±2.2	5.99±1.7	6.01±1.5	10.76±2.8	32.25	0.000
6 months	0.2±0.05	4.6±1.3	6.14±1.6	7.8±2.3	6.81±1.9	10.73±3.2	31.74	0.000
1 year	1.4±0.4	3.52±1.2	6.13±1.8	4.09±1.2	4.61±1.1	10.09±2.5	37.83	0.000
Overall	1.04±0.6	4.6±0.77	6.52±0.58	5.97±1.88	6.1±1.2	10.26±0.78	38.95	0.000

**Figure 8:**
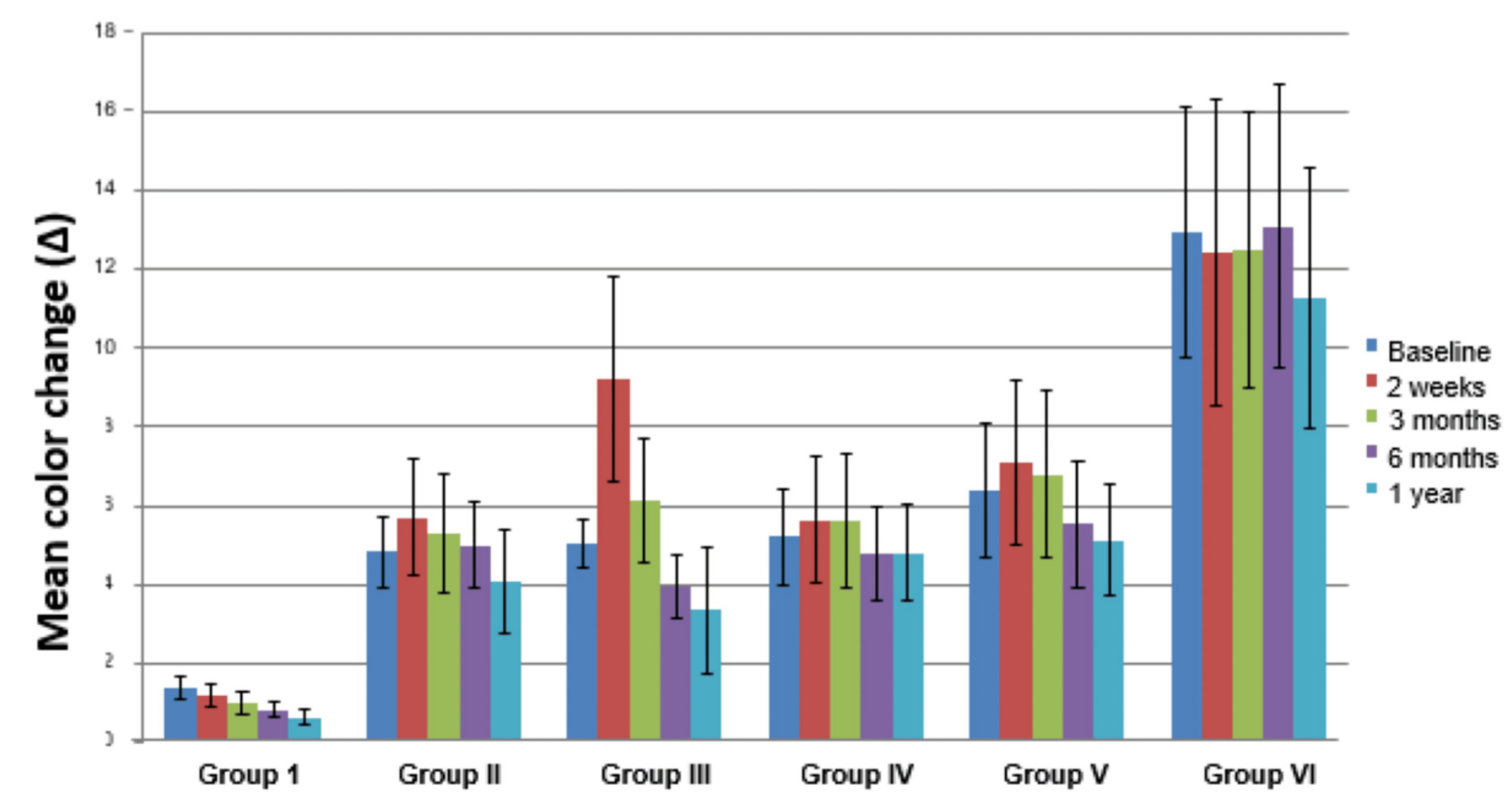
Mean color change (Δ) through treatment using the CIELAB system

Comparisons of differences in color measurement by digital image capturing and analysis using the CIELAB system between all the groups through all bleaching intervals were statistically highly significant. Group VI (Δ=12.44) showed significant improvement in shades of bleached teeth as compared to other groups (p=0.000) (Table 2, Figure 8).

**Table 2:** Color change (Δ) for all the bleaching groups at various intervals and overall effect on color change by digital image capturing and analysis using the CIELAB system *SD: standard deviation; ANOVA: analysis of variance

	Color change (Δ) for all the bleaching groups (Mean ± SD)						ANOVA	
Interval	Group I	Group II	Group III	Group IV	Group V	Group VI	F	p-value
Baseline	1.38±0.3	4.83±0.9	5.03±0.6	5.21±1.2	6.41±1.7	12.95±3.2	55.14	0.000
2 weeks	1.17±0.3	5.71±1.5	9.23±2.6	5.64±1.6	7.09±2.1	12.41±3.9	27.49	0.000
3 months	1.01±0.29	5.3±1.5	6.13±1.6	5.6±1.7	6.8±2.1	12.49±3.5	33.4	0.000
6 months	0.82±0.21	4.99±1.1	3.92±0.8	4.77±1.2	5.52±1.6	13.1±3.6	52.79	0.000
1 year	0.64±0.18	4.07±1.3	3.35±1.6	4.81±1.2	5.13±1.4	11.27±3.3	39.98	0.000
Overall	1.004±0.28	4.98±0.6	5.53±2.3	5.2±0.4	6.19±0.8	12.44±0.7	58.39	0.000

**Figure 9:**
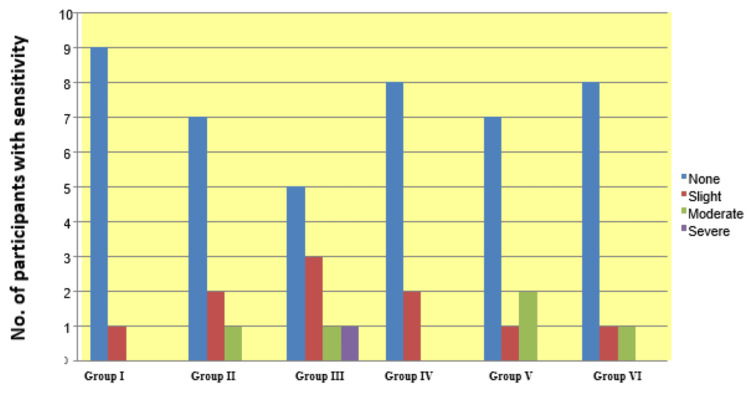
Dental sensitivity during bleaching

Patients’ dental sensitivity was higher at baseline after bleaching (with the typical peak within 24 hours after bleaching) as compared to two-week time intervals after bleaching, and the difference was not statistically significant (p > 0.05). Overall, 16 (26.6 %) participants experienced postoperative sensitivity. Five participants in group III experienced postoperative sensitivity. No sensitivity was recorded three months after treatment (Figure 9).

**Figure 10:**
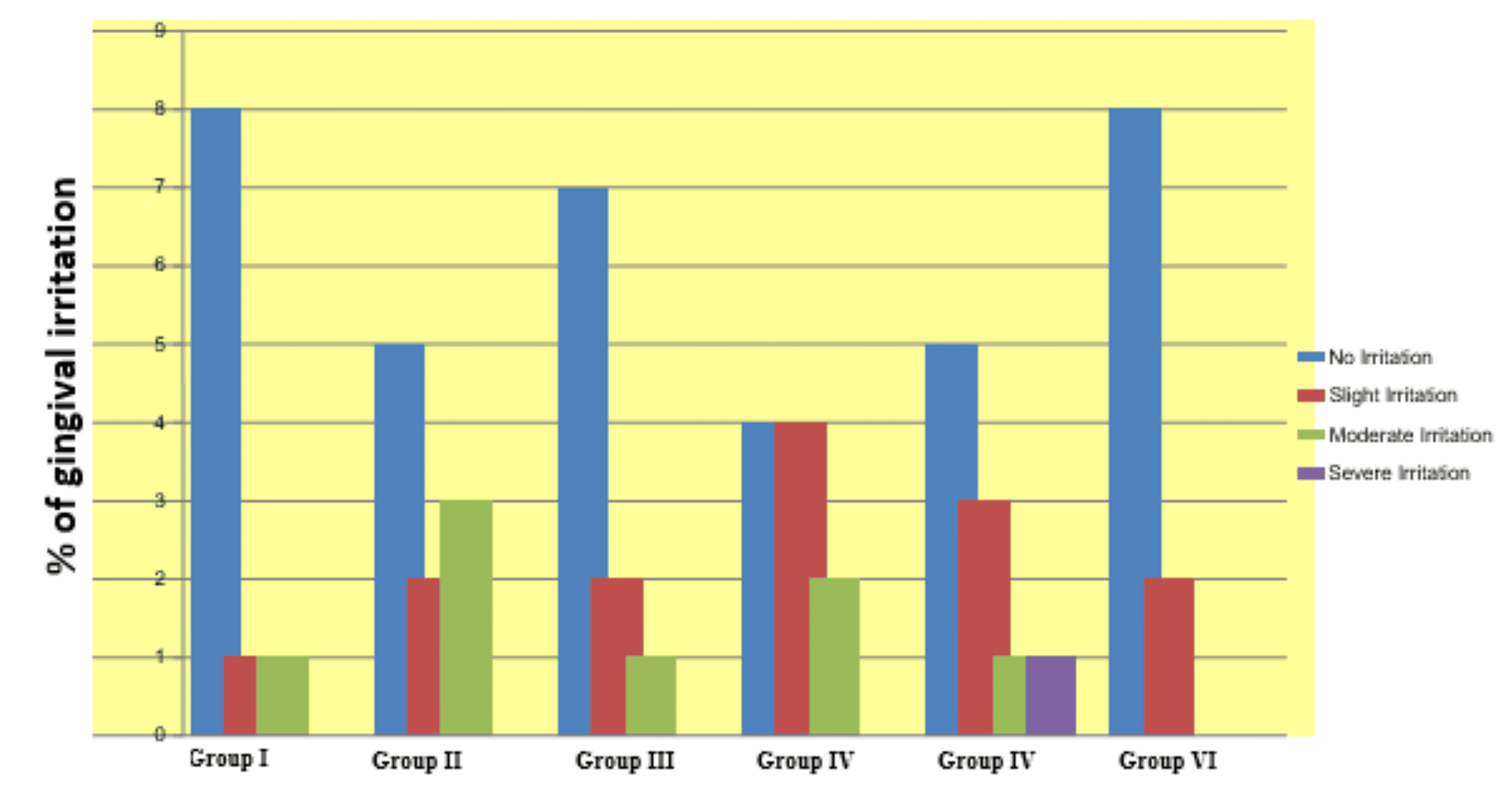
Gingival irritation during bleaching

There was no significant difference in gingival irritation between the bleaching groups. Twenty-three patients from all the groups showed gingival irritation while group IV recorded the maximum number of participants (n=6) with gingival irritation. Gingival irritation was peak at the baseline after bleaching as compared to two weeks, but the difference was not statistically significant (p > 0.05). No gingival irritation was recorded at three-month intervals (Figure 10).

Thirty-five of the 60 participants (58.3%) recorded that the treatment whitened their teeth “moderately” and “a lot” while seven participants (11.6%) recorded “a slight” difference. Nine participants from Group VI were highly satisfied with the bleaching treatment. Further, 6 patients from group III were also highly satisfied, whereas 18 participants (group I - 7, group II - 5, and group IV - 6) found no difference in tooth color (Table 3).

**Table 3:** Response of participants to the level of satisfaction with the treatment rendered n*: number of participants

Groups	Level of satisfaction with bleaching (n*)				X^2^	P-value
	None	Slight	Moderate	A lot		
Group I	7	1	1	1	28.2	0.000
Group II	5	2	2	1		
Group III	0	1	3	6		
Group IV	6	0	2	2		
Group V	0	3	3	4		
Group VI	0	0	0	9		

In response to the question, if they would recommend the rendered treatment to others, 27 participants answered “yes” and 17 persons answered “maybe” while 16 participants did not agree to recommend the treatment to others. Maximum participants from Group I denied recommending the treatment to other fellows (Table 4).

**Table 4:** Response of participants to the recommendation of the treatment to others n*: number of participants

Groups	Response to recommendation to others (n*)			X^2^	P-value
	Yes	Maybe	No		
Group I	1	3	6	21.9	0.001
Group II	2	3	5		
Group III	8	1	1		
Group IV	3	5	2		
Group V	4	4	2		
Group VI	9	1	0		

Tukey’s post-hoc analysis showed statistically significant color change at baseline and two-week, three-month, six-month, and one-year intervals. Nonetheless, the comparison of combined color change values showed highly significant differences between bleaching group VI (Δ=10.26) (Ti-ON 20% H2O2) and other bleaching groups: control group, 15% H_2_O_2_, 38% H_2_O_2_, 15% CP, and 35% CP where P < 0.05. This presented that, the in-office Ti-ON 20% H2O2 bleaching technique followed by 38% H_2_O_2_ has a significant bleaching effect (Δ=10.26 and Δ6.52) when compared to other bleaching techniques. The post-hoc comparison between Groups II and III, Group IV with Groups II and III, and Group V with Groups II, III, and IV were not significant. All the groups showed a significant color change as compared to Group I (Table 5).

**Table 5:** Means, standard deviations, and Tukey’s post-hoc analysis of color change (Δ) for all the bleaching groups through all bleaching intervals Non-significant: at P >0.05 Significant: at P < 0.05 P1 = significance between Group 1 and other groups P2 = significance between Group 2 and other groups P3 = significance between Group 3 and other groups P4 = significance between Group 4 and other groups P5 = significance between Group 5 and Group 6

Parameter	Group I	Group II	Group III	Group IV	Group V	Group VI
Mean	1.04	4.6	6.52	5.97	6.1	10.26
SD	0.6	0.77	0.58	1.88	1.2	0.78
P1		<0.001	<0.001	<0.001	<0.001	<0.001
P2			>0.05	>0.05	>0.05	<0.001
P3				>0.05	>0.05	<0.001
P4					>0.05	<0.001
P5						<0.001

## Discussion

Tooth appearance is an important factor defining beauty and attractiveness, and aesthetic dental treatments are highly demanded by patients nowadays. Tooth bleaching is one of the most conservative dental treatments to improve or enhance a person’s smile. There are three different bleaching methods: at home, in the office, and walking bleaching. The former two techniques are advocated for improving the color of discolored vital teeth [14]. Compared to the home bleaching agents, available office bleaching products generally contain relatively high levels of bleaching agents, for example, 35% H_2_O_2_, and are applied for shorter periods [15]. Factors contributing to the efficacy of office bleaching are temperature, H_2_O_2_ concentration, application time, light or energy sources, and the presence of some catalysts. A combination of these factors can accelerate the office bleaching process and result in a higher short-time efficacy [16].

The present study included 60 participants who were equally divided into six groups. For these groups, the bleaching systems selected were 15% H_2_O_2_, 38% H_2_O_2_, 15% CP, 35% CP, and Ti-ON 20% H_2_O_2_. Each system had a different formulation and concentration and was applied to each group by following the manufacturers’ guidelines. All the participants were evaluated for the effectiveness of different bleaching systems through color difference, dental hypersensitivity, gingival irritation, and patient satisfaction. To facilitate the comparison of color test results, Vita Bleached Guide 3D-Master (subjective method) and CIE L*a*b* system (subjective method) were used. The Vita Guide displays a greater emphasis on the extra light area of the tooth color space and the CIE L*a*b* system provides more details to distinguish minor color differences [17].

There is a general belief among the general population and anecdotal evidence among dentist practitioners that in-office bleaching is superior to at-home bleaching. Some manufacturers claim that high-concentration hydrogen peroxide bleaching agents are superior and faster compared to low-concentration carbamide peroxide at-home products [18]. Many studies have been done to evaluate the clinical behavior of different bleaching systems (in-office and at-home) evaluating the color change, postoperative sensitivity, gingival irritation, and patient satisfaction [11,14,18-20].

Regarding the color assessment under the conditions of the present study, the Ti-ON 20% H_2_O_2_ bleaching technique had a significant bleaching effect (Δ=10.26) when compared to other bleaching techniques. This was indicated by the increased value of color change Δ and ΔE=12.44. This result agreed with Kishi et al. who found that the addition of Ti-ON to the H_2_O_2_-based bleaching agent could enhance the bleaching efficacy of the bleaching agent when exposed to visible light. The Ti-ON-containing gel can accelerate the bleaching process, decreasing the clinical time required and potentially lowering the adverse effects of bleaching agents with longer application times on enamel [14].

The most acceptable explanation of these findings was that titanium dioxide has been known as the most important semiconductor photocatalyst reacting to visible light. Ti-ON was modified from the original titanium dioxide photocatalyst and the application of visible light on a bleaching agent containing H_2_O_2_ and Ti-ON could increase the bleaching efficacy. Ti-ON works as a photocatalyst reacting to visible light, especially at low wavelengths. It was suggested that bleaching was enhanced by hydroxyl radical generation through the photocatalytic action of Ti-ON. Furthermore, light-induced dehydration may have played a significant role in immediate bleaching efficacy.

The results of this study were in accordance with Suyama et al. and Suemori et al. [21,22]. From these studies, it was evident that the high-intensity halogen with Ti-ON and H_2_O_2_ caused the most significant reduction in stain concentration and the presence of Ti-ON as a photocatalyst had a remarkable effect on the bleaching and color change. Further, Asahi et al. revealed that the optical absorption and photocatalytic activity of nitrogen-doped titanium oxides improved under visible light (wavelength < 500 nanometers) in the photodegradation of methylene blue and gaseous acetaldehyde and hydrophilicity of the film surface [23].

Moreover, the results of this study showed that 38% H_2_O_2 _has a significant bleaching effect (ΔE=10.26 and ΔE=6.52) when compared with other peroxides concentrations. These results agreed with Sulieman et al. who found that the concentration of hydrogen peroxide in a bleaching gel had an obvious effect on the number of applications required to produce an optimal shade outcome [24]. The findings of the present study were contradicted by McCaslin et al. who used 10% carbamide peroxide gel in a bleaching tray at night [25]. However, this discrepancy may be attributed to the fact that all concentrations could produce an evident bleaching effect if used for a longer duration [26].

However, the findings of the present study were contradictory to Zekonis et al., who found that the in-office treatment was not superior in tooth whitening to the at-home treatment [18], and Giachetti et al., who found no clinically significant difference in bleaching efficacy between power and at-home bleaching systems [27]. This disagreement is justified by the different methods of study (time of evaluation, bleaching agents used, time of application, bleaching agent pH, and usage of fluoride). Also, this disagreement in results may be attributed to the prolonged contact between the bleaching agent in at-home bleaching systems and the teeth compared to a lower number of bleaching sessions and the duration of the bleaching gel application of in-office bleaching systems. However, a study found a novel idea of combining home and in-office bleaching with 16% carbamide peroxide to be more effective in color changes [28].

The most feasible explanation for these findings was that the diffusion of a liquid into a substrate is governed by Fick's law, which relates to small molecules, such as water, the bleaching gel would hinder the diffusion process [29]. However, it may be argued that this represents a very simplistic approach to the interaction of bleaching complex interactions between tooth and bleaching material that involves diffusion and reaction of the peroxide moieties with the chromogens.

Regarding teeth hypersensitivity, 23% of all the patients showed postoperative hypersensitivity with higher sensitivity recorded for Group III. The sensitivity was experienced immediately after bleaching (with the typical peak within 24 hours after bleaching) as compared to two weeks after bleaching. No sensitivity was recorded three months after treatment. The results were close to that of Pontes et al., Jorgensen et al., and Haywood et al. with 15-20% of patients reporting sensitivity during vital bleaching [30-32]. The results disagreed with the findings of Tam et al. and Leonard et al. who reported a higher incidence of tooth sensitivity [33,34]. The development of tooth sensitivity can be a multifactorial phenomenon. If the whitening tray is fabricated from a thick material, it can result in an appliance producing minor orthodontic movements. Allergies and chemical sensitivity to the composition of the tray or the bleaching gel. And the free radical formation of the whitening gel. It can also result from overzealous tooth brushing during participation in a clinical trial. Glycerin, which is used to carry the active ingredient, can absorb water and therefore can have a dehydration effect, hence resulting in sensitivity [34].

Regarding gingival irritation, there was no significant difference between the preoperative gingival index scores and those recorded at any point during the study. Thirty-five percent of the patients for all groups showed gingival irritation (mild-moderate). Higher gingival irritation was recorded for bleaching with group IV 15% carbamide peroxide. Gingival irritation was higher immediately (at baseline) after bleaching but no gingival irritation was recorded at three-month intervals after treatment. Martini et al. and Ferraz et al. also found low gingival irritation in their respective studies on the use of different bleaching systems [10,35].

Evaluation of participants' satisfaction showed that 35 participants (85.5%) met with a “moderate” to “a lot” level in whitening while seven participants (11.6%) recorded “a slight” difference. There was a significant difference in participant satisfaction between all bleaching groups with Group VI followed by Group III recording the highest satisfaction scores.

This can be attributed to the high concentration of H_2_O_2_ in both the bleaching systems. The usage of Ti-ON as a photocatalyst showed the highest color change (ΔE). The present findings disagreed with those of Marson et al. who concluded that there was no difference in color change and participants' satisfaction between groups of in-office bleaching treatments with and without the use of light-activation sources [36]. This discrepancy between these results may be due to the differences in attitudes and perceptions toward dental appearance that differ among populations and among individuals in a population. Satisfaction with dental appearance is determined by cultural factors and individual preferences, varying between individuals and cultures and changing over time [37]. Goettems ML et al. studied oral health-related quality of life and found significant satisfaction irrespective of bleaching techniques [38].

As in most tooth bleaching clinical studies, limitations include participants being mostly between 20 and 30 years of age, probably due to the inclusion/exclusion criteria requiring anterior teeth free of decay/restorations and the higher demand for aesthetic treatments by younger individuals. Although evidence suggests a significant relationship between the subject’s age and the magnitude of the whitening response (younger subjects experience greater effects), in older populations, tooth bleaching could be effective considering the reported positive correlation between yellow hues and bleaching effects [7].

The merit of the present study was the number of participants who used five different bleaching systems along with a control group. Few studies conducted randomized control trials with more than two bleaching systems [39-42]. The present study used potassium nitrate products, such as Ultra EZ, immediately after treatment and for 15 minutes to 1 hour per day until sensitivity subsided. A study on the management of post-bleaching tooth sensitivity found similar effects with the use of either sodium fluoride varnish and/or diode laser [43].

## Conclusions

The use of in-office and at-home bleaching systems with different concentrations of hydrogen peroxide and carbamide peroxide shows excellent results; however, their use has raised questions regarding color changes, clinical behavior, and adverse effects on the tooth structure. This study was carried out to evaluate the effects of different bleaching products on the clinical behavior of five bleaching systems. From the present study, it is evident that there is a development of dental sensitivity and gingival inflammation irrespective of the bleaching system used. The color assessment revealed that either system gave the desired result but had different levels of patient satisfaction. Yet, the overall outcome of GC Ti-On 20% H_2_O_2_ had the best results among clinical parameters and patient satisfaction.

It would be valuable to suggest potential avenues for future research based on the findings of this study and to emphasize the need for continued investigation into the long-term effects and comparative efficacy of different bleaching systems.

## References

[1] Alkhatib MN, Holt R, Bedi R (2005). Age and perception of dental appearance and tooth colour. Gerodontology.

[2] Watts A, Addy M (2001). Tooth discolouration and staining: a review of the literature. Br Dent J.

[3] Joiner A (2004). Tooth colour: a review of the literature. J Dent.

[4] Nathoo SA (1997). The chemistry and mechanisms of extrinsic and intrinsic discoloration. J Am Dent Assoc.

[5] Yuji S, Masayuki O, Shinichiro O, Ryuzo K, Junji T, Masaomi I, Cho T (2009). Effects of light sources and visible light-activated titanium dioxide photocatalyst on bleaching. Dent Mater J.

[6] Paes Leme AF, dos Santos JC, Giannini M, Wada RS (2004). Occlusion of dentin tubules by desensitizing agents. Am J Dent.

[7] Joiner A (2006). The bleaching of teeth: a review of the literature. J Dent.

[8] Matis BA, Cochran MA, Eckert G, Carlson TJ (1998). The efficacy and safety of a 10% carbamide peroxide bleaching gel. Quintessence Int.

[9] Attin T, Hannig C, Wiegand A, Attin R (2004). Effect of bleaching on restorative materials and restorations--a systematic review. Dent Mater.

[10] Martini EC, Favoreto MW, Coppla FM, Loguercio AD, Reis A (2020). Evaluation of reservoirs in bleaching trays for at-home bleaching: a split-mouth single-blind randomized controlled equivalence trial. J Appl Oral Sci.

[11] Ontiveros JC, Paravina RD (2009). Color change of vital teeth exposed to bleaching performed with and without supplementary light. J Dent.

[12] Sa Y, Chen D, Liu Y, Wen W, Xu M, Jiang T, Wang Y (2012). Effects of two in-office bleaching agents with different pH values on enamel surface structure and color: an in situ vs. in vitro study. J Dent.

[13] Haywood VB (1992). History, safety, and effectiveness of current bleaching techniques and applications of the nightguard vital bleaching technique. Quintessence Int.

[14] Kishi A, Otsuki M, Sadr A, Ikeda M, Tagami J (2011). Effect of light units on tooth bleaching with visible-light activating titanium dioxide photocatalyst. Dent Mater J.

[15] Haywood VB, Heymann HO (1989). Nightguard vital bleaching. Quintessence Int.

[16] Sulieman M, Addy M, Macdonald E, Rees JS (2004). A safety study in vitro for the effects of an in-office bleaching system on the integrity of enamel and dentine. J Dent.

[17] Paravina R (2008). New shade guide for tooth whitening monitoring: visual assessment. J Prosthet Dent.

[18] Zekonis R, Matis A, Carlson J (2003). Clinical evaluation of in-office and at-home bleaching treatments. Oper Dent.

[19] Matis BA, Cochran MA, Wang G, Eckert GJ (2009). A clinical evaluation of two in-office bleaching regimens with and without tray bleaching. Oper Dent.

[20] Ontiveros JC, Eldiwany MS, Paravina R (2012). Clinical effectiveness and sensitivity with overnight use of 22% carbamide peroxide gel. J Dent.

[21] Suyama Y, Otsuki M, Ogisu S (2009). Effects of light sources and visible light-activated titanium dioxide photocatalyst on bleaching. Dent Mater J.

[22] Suemori T, Kato J, Nakazawa T, Akashi G, Igarashi A, Hirai Y (2008). Effects of light irradiation on bleaching by a 3.5% hydrogen peroxide solution containing titanium dioxide. Laser Phys.

[23] Asahi R, Morikawa T, Ohwaki T, Aoki K, Taga Y (2001). Visible-light photocatalysis in nitrogen-doped titanium oxides. Science.

[24] Sulieman M, Addy M, MacDonald E, Rees JS (2004). The effect of hydrogen peroxide concentration on the outcome of tooth whitening: an in vitro study. J Dent.

[25] McCaslin AJ, Haywood VB, Potter BJ, Dickinson GL, Russell CM (1999). Assessing dentin color changes from nightguard vital bleaching. J Am Dent Assoc.

[26] Leonard RH, Sharma A, Haywood VB (1998). Use of different concentrations of carbamide peroxide for bleaching teeth: an in vitro study. Quintessence Int.

[27] Giachetti L, Bertini F, Bambi C, Nieri M, Scaminaci Russo D (2010). A randomized clinical trial comparing at-home and in-office tooth whitening techniques: a nine-month follow-up. J Am Dent Assoc.

[28] Knezović Zlatarić D, Žagar M, Illeš D (2019). A clinical study assessing the short-term efficacy of combined in-office/at-home whitening treatment. J Esthet Restor Dent.

[29] Braden M (1976). Biophysics of the tooth. Front Oral Physiol.

[30] Pontes M, Gomes J, Lemos C, Leão RS, Moraes S, Vasconcelos B, Pellizzer EP (2020). Effect of bleaching gel concentration on tooth color and sensitivity: a systematic review and meta-analysis. Oper Dent.

[31] Jorgensen MG, Carroll WB (2002). Incidence of tooth sensitivity after home whitening treatment. J Am Dent Assoc.

[32] Haywood VB, Caughman WF, Frazier KB, Myers ML (2001). Tray delivery of potassium nitrate-fluoride to reduce bleaching sensitivity. Quintessence Int.

[33] Tam L (1999). Clinical trial of three 10% carbamide peroxide bleaching products. J Can Dent Assoc.

[34] Leonard RH Jr, Bentley C, Eagle JC, Garland GE, Knight MC, Phillips C (2001). Nightguard vital bleaching: a long-term study on efficacy, shade retention. side effects, and patients' perceptions. J Esthet Restor Dent.

[35] Ferraz LN, Vieira I, Ambrosano GM, Lopes MA, Lima DA (2022). Effect of bleaching gels with different thickeners under normal and hyposalivation conditions: in situ study. J Appl Oral Sci.

[36] Marson FC, Sensi LG, Vieira LC, Araújo E (2008). Clinical evaluation of in-office dental bleaching treatments with and without the use of light-activation sources. Oper Dent.

[37] Tin-Oo MM, Saddki N, Hassan N (2011). Factors influencing patient satisfaction with dental appearance and treatments they desire to improve aesthetics. BMC Oral Health.

[38] Goettems ML, Fernandez MD, Donassollo TA, Henn Donassollo S, Demarco FF (2021). Impact of tooth bleaching on oral health-related quality of life in adults: a triple-blind randomised clinical trial. J Dent.

[39] Ahrari F, Akbari M, Mohammadipour S, Fallahrastegar A, Sekandari S (2020). The efficacy and complications of several bleaching techniques in patients after fixed orthodontic therapy. A randomized clinical trial. Swiss Dent J.

[40] Mushashe AM, Coelho BS, Garcia PP, Rechia BN, da Cunha LF, Correr GM, Gonzaga CC (2018). Effect of different bleaching protocols on whitening efficiency and enamel superficial microhardness. J Clin Exp Dent.

[41] Pereira R, Silveira J, Dias S, Cardoso A, Mata A, Marques D (2022). Bleaching efficacy and quality of life of different bleaching techniques - randomized controlled trial. Clin Oral Investig.

[42] Pinzan-Vercelino CR, Lima SN, Pereira FF, Gurgel JA, Silva GR, Freitas KM (2022). Efficacy of products for bleaching and whitening under orthodontic brackets. Dental Press J Orthod.

[43] Yahya G, AlAlwi A, Shurayji F, Baroom W, Rajeh M, AbdelAleem N (2022). Effectiveness of sodium fluoride varnish and/or diode laser in decreasing post-bleaching hypersensitivity: a comparative study. Saudi Dent J.

